# *OsDREB2A*, a Rice Transcription Factor, Significantly Affects Salt Tolerance in Transgenic Soybean

**DOI:** 10.1371/journal.pone.0083011

**Published:** 2013-12-20

**Authors:** Xiu-xiang Zhang, Yu-juan Tang, Qi-bin Ma, Cun-yi Yang, Ying-hui Mu, Hai-cui Suo, Lai-hui Luo, Hai Nian

**Affiliations:** 1 The Guangdong Subcenter of National Center for Soybean Improvement, State Key Laboratory of Agricultural and Biological Resources Protection and Utilization in Subtropics, College of Agriculture, South China Agricultural University, Guangzhou, China; 2 School of Life Science, Jiaying University, Meizhou, China; University of Arizona, United States of America

## Abstract

The dehydration responsive element binding (DREB) transcription factors play an important role in regulating stress-related genes. OsDREB2A, a member of the DREBP subfamily of AP2/ERF transcription factors in rice (*Oryza sativa*), is involved in the abiotic stress response. *OsDREB2A* expression is induced by drought, low-temperature and salt stresses. Here, we report the ability of *OsDREB2A* to regulate high-salt response in transgenic soybean. Overexpressing *OsDREB2A* in soybeans enhanced salt tolerance by accumulating osmolytes, such as soluble sugars and free proline, and improving the expression levels of some stress-responsive transcription factors and key genes. The phenotypic characterization of transgenic soybean were significantly better than those of wild-type (WT). Electrophoresis mobility shift assay (EMSA) revealed that the OsDREB2A can bind to the DRE core element *in vitro*. These results indicate that OsDREB2A may participate in abiotic stress by directly binding with DRE element to regulate the expression of downstream genes. Overexpression of *OsDREB2A* in soybean might be used to improve tolerance to salt stress.

## Introduction

Adverse environmental conditions, such as high-salt and drought, affect plant growth and productivity. To survive and adapt in adverse conditions, plants have evolved a variety of stress response mechanisms at the molecular, cellular, physiological, and biochemical levels. Transcription factors play an important role in upstream gene regulation of plant stress response pathways [1]. Among these are the AP2/EREBP transcription factors identified in a variety of higher plants, such as *Arabidopsis*, *Nicotiana tabacum*, *Solanum lycopersicum L.*, *Oryza sativa*, *Zea mays L.*, *Ricinus communis L.*, and *Brassica* [2]–[4]. AP2/EREBP transcription factor genes are involved in many plant functions including growth, hormone signal transduction, pathogen responses, and responses to stresses such as drought and salt. They are characterized by the presence of the highly conserved AP2/EREBP DNA-binding domain of about 58 or 59 amino acid residues, additionally the AP2/ERF domains bind to the dehydration responsive element (DRE) or GCC-box [3], [5]. Both dehydration responsive element binding (DREB) and ethylene responsive factors (ERF) subfamilies are of particular interest due to their involvement in stress response. The genes in the DREB subfamily play a crucial role in the resistance of plants to abiotic stresses by recognizing DRE with a core motif of A/GCCGAC [6], [7]. The ERF subfamily includes a large number of ERFs [8], [9], which are mainly involved in the plant response to biotic stresses like pathogenesis by recognizing the *cis*-acting element AGCCGCC, known as the GCC box [8], [10]. Of all the subfamilies, DREBs can regulate expression of downstream genes by binding to *cis*-acting DRE/CRT elements located in the promoter region of downstream stress-responsive genes. An increasing number of *DREB* genes have been cloned from various plants [11]. *DREB* genes cloned from *Oryza sativa* include *OsDREB1A* to *OsDREB1G*, *OsDREB2A*, and *OsDREB2B*, although only *OsDREB1A*, *OsDREB1E*, *OsDREB1G*, *OsDREB2A*, and *OsDREB2B* can specifically bind to DRE elements [12]–[14]. DREB2A/CBF exist in a wide array of plants, in addition to *Atriplex hortensis* [15], including plants that are acclimatized to salt, such as perennial *Lolium perenne* [16], [17]. Overexpressions of *DREB orthologs* in *Arabidopsis* and *Oryza sativa* result in improved resistance to abiotic stresses, such as salt and drought [6], [12], [13].

The cDNAs encoding DRE-binding protein, *DREB2A*, have been isolated by using yeast one-hybrid screening in *Arabidopsis* [7]. A *OsDREB* homologous gene, *OsDREB2A*, which was isolated from rice classified into A-2 subgroup in DREB subfamily [12]. Overexpression of rice *OsDREB2A* in transgenic *Arabidopsis* also enhanced tolerance to dehydration and high-salt stress [12], [18]. To further analyze whether *OsDREB2A* has a function in abiotic stress in *Glycine max*, we overexpressed *OsDREB2A* in soybeans and investigated salt-stress tolerance in seeds and seedlings in present study. Taken together, our results will be helpful in determining the functions of *OsDREB2A* in different species.

## Materials and Methods

### Rice Seeds and Salt Treatment of Rice Seedlings

Rice seeds of 9311 were germinated on vermiculite in a light chamber at 25°C for 3 weeks. To determine expression pattern of *OsDREB2A* gene under high-salt stress, 3-week-old seedlings were treated with 200 mM NaCl, and samples were collected at 0 h, 6 h, 12 h, 24 h, 48 h and 72 h.

### Isolation of *OsDREB2A*

The extremely high sequence homology (99% at the nucleotide level) suggests that the *OsDREB1* (accession no. AY064403) cloned belongs to the same cluster with the *OsDREB2A* (accession no. JQ341059) at nucleotide level. The full-length cDNA of *OsDREB1* was amplified by RT-PCR from leaves of rice using gene-specific primers (F: 5′-CTGATAGCCTCCTTGATTTT-3′, R: 5′-AAGACGAAAACCGTAAATG-3′) (accession no. AY064403). The opening reading frame (ORF) was 846 bp, ligated into pMD-18T vector by T4 DNA ligase and sequencing confirmation.

### Construction of *OsDREB2A* Expression Vector

An 846 bp coding region of *OsDREB2A* was amplified from the *OsDREB2A*-T vector by PCR. The primers were designed as F: 5′-GGATCCATGCTGTTTCGATTTGTG-3′ and R: 5′-GGTACCCTAATAGGAGAAAAGGCT-3′ (accession no. JQ341059) with the *Bam*H I and *Kpn* I sites, respectively. After sequencing confirmation, the coding region of *OsDREB2A* was digested with *Bam*H I/*Kpn* I and was cloned into the GUS position of the intermediate vector of pUC18-pZY102 (The plasmids pUC18 (TaKaRa, Dalian, China) deleted the sites between *Bam*H I and *Kpn* I and the segment of pZY102 with 35S-GUS-NOS sequence was digested with restriction endonuclease *Hin*d III, and then the two linearized parts were linked together, named as pUC18-pZY102). After sequencing confirmation, the segment of 35S-*OsDREB2A*-NOS from pUC18-pZY102 was inserted into pZY101 vector at *Hin*d III site, which was named pZY101-*OsDREB2A*. The resulting binary vector was introduced into *Agrobacterium tumefaciens* strain EHA101 by the freeze-thaw method [19], which was then used for further genetic soybean transformation.

### Soybean Transformation

Mature soybean seeds of cultivar Huachun 3 bred in Guangdong Subcenter of National Center for Soybean Improvement were surface sterilized for 13.5 h using chlorine gas produced by mixing 4.2 ml of 12 N HCl with 100 ml sodium hypochlorite in tightly sealed desiccators [20]. The cotyledonary node method described previously was used [21]. T3 generation homozygous lines of soybeans were used for the phenotype analysis.

### High-salt Tolerance Characterization

For the germination assay, 18 seeds for each transgenic soybean or WT were surface sterilized and placed on half-strength Murashige and Skoog (MS) [22] agar plates containing different concentrations of NaCl (0, 200, 250, and 300 mM) or ABA (0, 1.0, 1.5, and 2.0 µM). Plates were placed in the growth chamber at constant 25°C and 16 h daily exposure to 1000 lux Grolux light for germination for 3 d. For salt germination assay, obvious differences in the phenotypes of the transgenic and WT seedlings were observed. For ABA germination assay, germination rates were then measured daily for one week. The WT seedlings were used as control. Each treatment had 3 replicates.

For hydroponic solution and soil culture experiments according to the previously described method by Angkinand *et al*. [23], with some slight modification. Briefly, the seeds were cultivated in silica sands in artificial climate incubator, for 25°C /28°C at intervals of 12 hours for 7 days. The seedlings were transplanted in Hoagland’s solution. The phenotypic characterizations were assayed in the seedling with ternately compound leaves, within 48 h after treatment with 300 mM NaCl.

In the soil culture test, transgenic and WT seedlings were grown in sterilizing soil (Jiangmei Horticultural Company, Shanghai, China) supplemented for 4 weeks under normal condition. We irrigated the transgenic and WT plants with 200 mM and 300 mM NaCl three times a week for two consecutive weeks, respectively.

### Free Proline and Soluble Sugars Concentration Determinations

For measuring of free proline and soluble sugars contents, the WT and transgenic plants were grown in pots under 16 h light at 28°C and 8 h dark at 22°C. Four-week-old plants were irrigated with 300 mM NaCl three times in one week. At the 12th day after the treatment, free proline and soluble sugar contents of leaves were measured as described previously by Saltzmann *et al*. [24].

### Gene Expression Analysis

The total RNAs were extracted using Trizol® reagent (Invitrogen, USA) from the six-week-old soybean seedlings of transgenic and WT, separately. After RNase-free DNase (TaKaRa, Dalian, China) treatment, approximately 1 µg total RNA was used for reverse transcription using the oligo (dT) primer and MMLV (Invitrogen, USA). qRT-PCR was performed using CFX96 (Bio-Rad, USA) and SYBR Green I (Bio-Rad, USA). Each of the cDNA samples was subjected to a qRT-PCR analysis in triplicate. The data were normalized using the reference gene *β-tubulin*. The relative expressions of specific genes were quantified using the 2^−△△Ct^ calculation. The primer pairs used for qRT-PCR are listed in Table 1.

**Table 1:** Primers used in this article.

Gene	Accession number	Primer sequence
*β-tubulin*	NM _178014	F:5′-CCTCGTTCGAATTCGCTTTTTG-3′R:5′-CAACTGTCTTGTCGCTTGGCAT-3′
*GmNHX1*	NM_001250237	F:5′- GTGCCTTGCTTACGACT -3′R:5′- GGTGAGCCAGGTTCTAC-3′
*GmDREB1*	NM_001250325	F:5′- CGGGTTTAGGAGATTGT-3′R:5′- TATTCCTCTGTATGGCTTC-3′
*GmDREB3*	NM_001251571	F:5′- GCAAATGGGTATCCGAAAT -3′R:5′- GGCCACGTAAGCAGAACA-3′
*GmDREB5*	EF583447	F:5′-GCCATTGTTTAGGCTTCC -3′R:5′- AAGGCTTCTCGGTCGTAG-3′
*GmDREB6*	EF551166	F:5′-ACTCCCTCTACCTCCTCTTC -3′R:5′- TTTCGGATACCCATTTGC-3′
*GmERF3*	EU681278	F:5′- GTCTACGGCAGCGAAAT -3′R:5′- CCAAGCCAGACACGAAC -3′
*GmERF7*	JN416602	F:5′- ATCTCCGACTTCATTCC-3′R:5′- CTCTGAACCCTGCCTC-3′
*Gmcor47-like*	NM_001253177	F:5′-GGCAGACGAGACCCAGAACA-3′R:5′-CTTTTTGAAACTCGGTGGCG-3′
*GmKin*	NM_001250504	F:5′-AATCTTGTGCTCGCTTAC-3′R:5′-TCCGCATTTGGGCTTTAT-3′
*GmP5CS*	NM_001251224	F:5′-TTGGGACTGCTGTGGT-3′R:5′-CAACTTGCGGCTTCTG-3′

### Electrophoretic Mobility Shift Assay (EMSA)

Glutathione-S-transferase (GST) fusion proteins preparation and gel mobility shift assay were conducted. An 846 bp fragment of *OsDREB2A* containing DNA-binding domain was amplified using the primer pair: *OsDREB2A*-F: 5′- GGATCCATGCTGTTTCGATTTGTGTCTTGC-3′; *OsDREB2A*-R: 5′- CTCGAGCCCAAATCGGAAAAGAGGATAATC-3′ (accession no. JQ341059). *Bam*H I and *Xho* I recognition sites were introduced and underlined. This fragment was cloned into the *Bam*H I/*Xho* I sites of the pGEX-4T-2 vector (Amersham Biosciences, Piscataway, USA) and transformed into *Escherichia coli* BL21 cells (Amersham Biosciences, Piscataway, USA) to produce the GST-fusion protein. The GST-fusion protein was purified using a glutathione Sepharose 4B column according to the manufacturer’s instruction (Amersham Biosciences, Piscataway, USA). The following double-stranded oligonucleotides were synthesized as WT or mutated (m) DRE element in EMSA: DRE forward, 5′-ATTTCATGGCCGACCTGCTTTCATGGCCGACCTGCTT-3′; and mGCC forward, 5′-ATTTCATGGAAGACCTGCTTTCATGGAAGACCTGCTT-3′. The gel mobility shift assay was conducted as described previously [7].

### Promoter Analysis

The promoter fragments of 10 abiotic stress-response genes were isolated from the soybean genome by searching the GmGDB database (http://www.plantgdb.org/GmGDB/), and *cis*-elements in promoters were searched in the PLACE database (http://www.dna.affrc.go.jp/PLACE/) [12], [18].

### Statistical Analysis

All data were processed by analysis of SPSS13.0 Data Editor (SPSS Inc., Chicago, Illinosis, USA). Values of *P*<0.05 were considered to be statistically significant.

## Results

### Overexpression of *OsDREB2A* Increase Tolerances to High-salt Stress in Transgenic Soybeans

**Figure 1:**
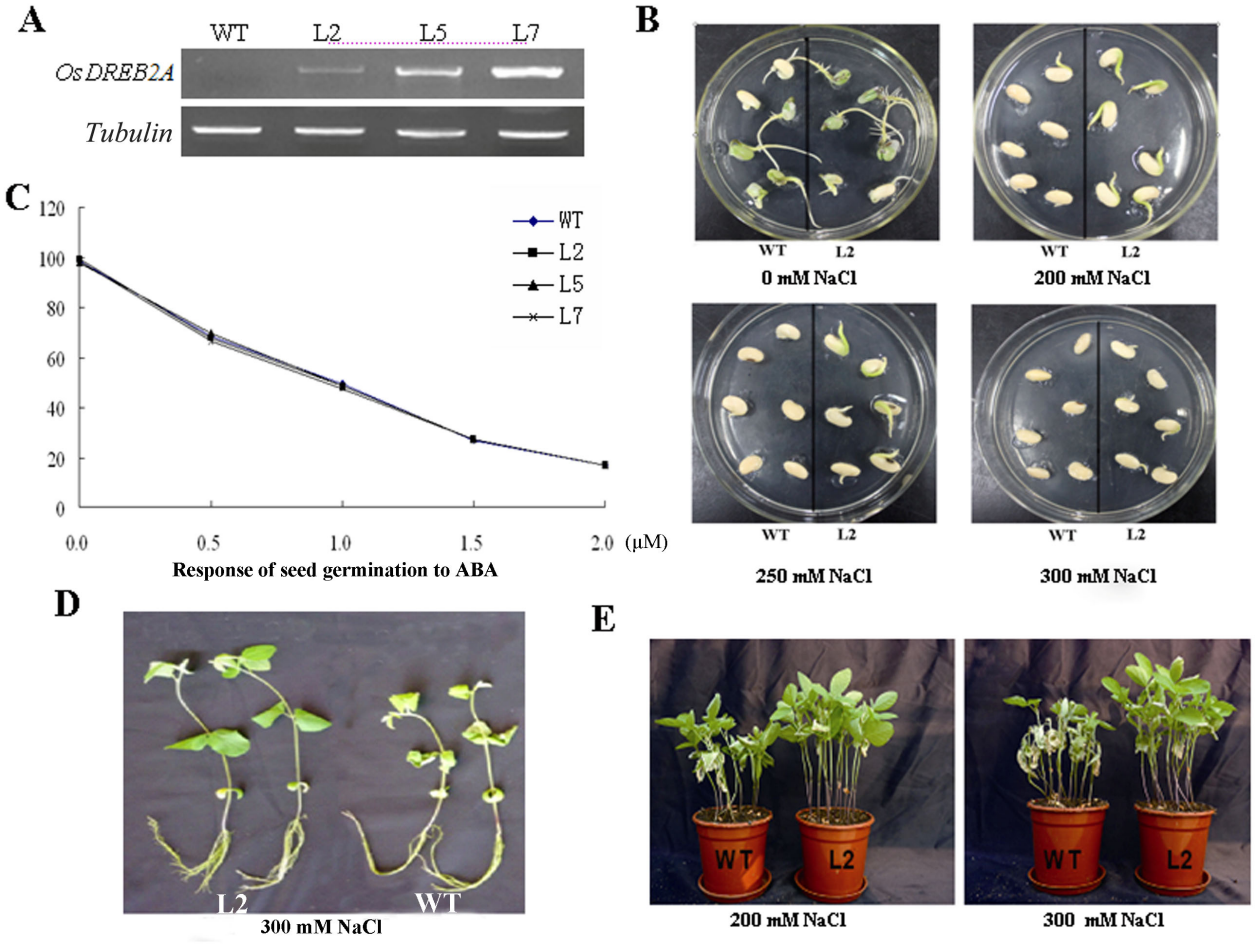
Response to salt stress of *OsDREB2A* overexpressing in plants. (A) *OsDREB2A* overexpression in soybean. Transcripts of *OsDREB2A* in transgenic lines (L2, L5, L7) were detected using RT-PCR, WT were used as control. *Tubulin* was used as an internal control and added 1 µg in 20 ul RT-PCR system. (B) Salt tolerances of germination assay. Eighteen seeds from the WT and transgenic lines were germinated on MS agar medium containing different concentrations of NaCl. The representative pictures were taken on the 7^th^ day. (C) Response of seed germination to ABA. Seeds on 1/2 MS solid medium with different concentrations of ABA were incubated at 25°C for 3 d and scored. (D) For hydroponic culture experiment, the taproot, lateral root and fibrous root were assayed between transgenic soybean (L2) and WT at 48 h after treatment with 300 mM NaCl, respectively. (E) Representative images of transgenic lines grown in soil under salt conditions. Four-week-old plants growing in soil irrigated with 200 mM NaCl and 300 mM NaCl three times a week for two consecutive weeks, respectively. The photographs were taken 14 d after salt treatment.

Overexpressing of *OsDREB2A* was done to examine the biological function in transgenic soybeans. RT-PCR analysis showed that *OsDREB2A* mRNA accumulated in all transgenic lines, but not in WT plants (Figure 1A). To examine the effects of the overexpression of *OsDREB2A* on salt tolerance, transgenic and WT seedlings were grown in normal and high-salt conditions. In the germination assays under normal conditions, no obvious differences in the phenotypes of the transgenic and WT seedlings were observed. After incubation in 200 mM NaCl, the germination rate of transgenic seeds was significant higher than that of WT seedlings (100% against 33.3%), but discernible phenotypic differences were observed. After incubation in 250 mM NaCl, the growth of the WT seedlings were completely inhibited, while, the bulk transgenic seedlings remained green and continued to grow, and the germination rate was 100%. After incubation in 300 mM NaCl, the transgenic seedlings began to show a lack of greening (Figure 1B). The germination of both WT and transgenic seedlings showed a similar sensitivity to exogenous ABA (Figure 1C).

Post-development assays were used as a growth indicator using high concentration of NaCl in both hydroponic solution and soil culture experiments. In the hydroponic solution assay, we found that the taproot had no obvious difference in circumference along the plant’s longitudinal axis between transgenic soybean and WT, but the transgenic lines had more branching lateral and fibrous root system than WT (Figure 1D), and the leaves of WT gradually became withered and turned yellow after incubation with 300 mM NaCl. In the soil culture experiment, the results showed that the leaves of WT plants became brown, wilted and curled up, in contrast, the transgenic leaves exhibited healthy growth under the treatment of 200 mM NaCl. However, under the treatment of 300 mM NaCl, most of WT leaves began to albino, and the transgenic leaves began to show a lack of greening (Figure 1E).

### Physiological Changes in *OsDREB2A* Transgenic Soybean

**Figure 2:**
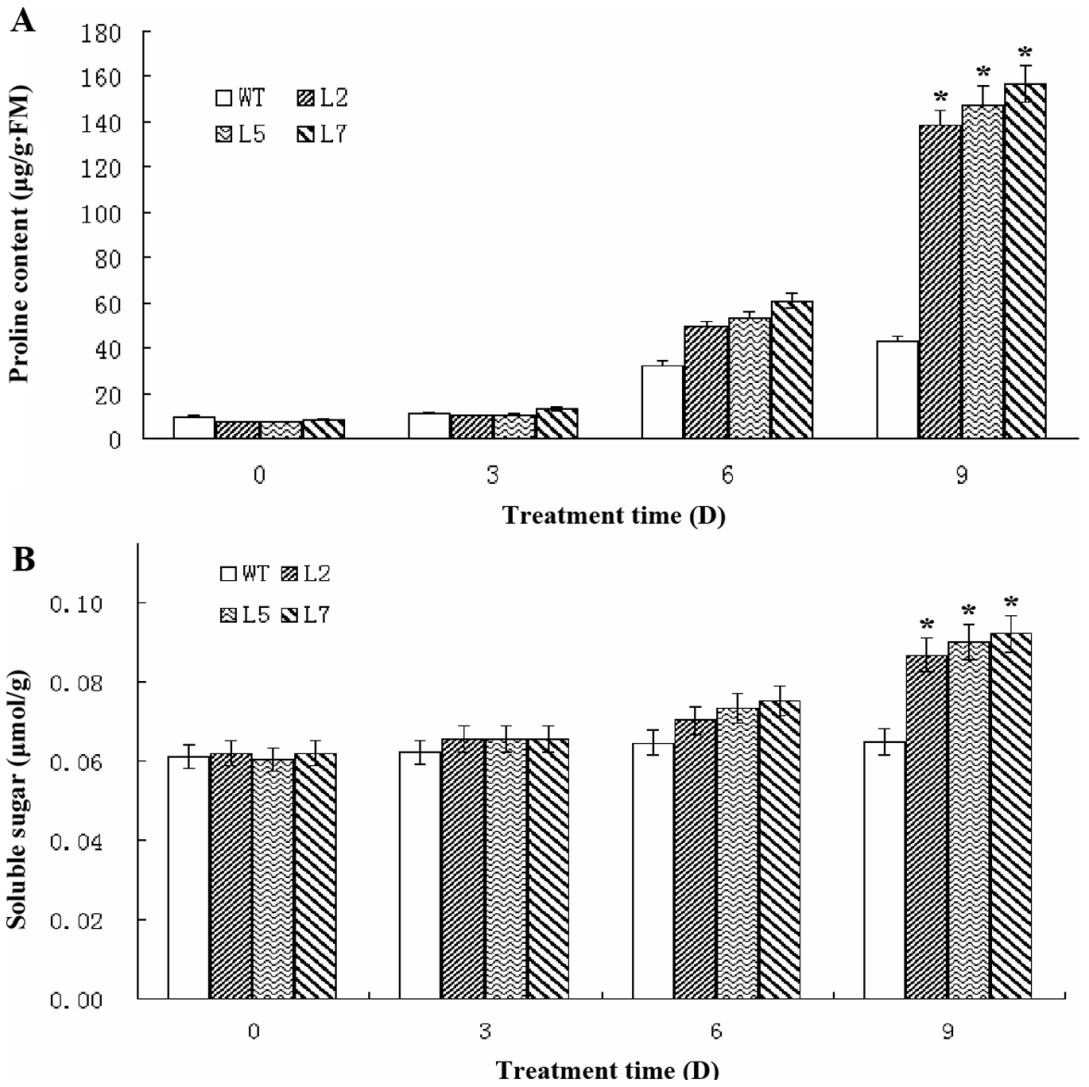
Measurement of soluble sugars and proline content of transgenic and WT plants after treatment with 300 The WT and transgenic lines (L2, L5 and L7) were grown in pots under normal condition for 4 weeks and then leaves of plants were harvested as controls. Then, the WT and transgenic soybeans were treated with 300 mM NaCl three times with 48 hours interval, while leaves were harvested at 0, 3rd, 6th and 9th days for free proline and soluble sugars content analysis, Data represented the average of three replicates ± SE. *indicated significant difference in comparison to the WT at *P*<0.05, respectively.

To evaluate physiological changes of transgenic soybean, contents of free proline and soluble sugars were measured following high-salt treatment. Under normal conditions, there were no evident morphological differences between the transgenic lines and WT in terms of their germination rates, seedling sizes, flowering times, primary root lengths and lateral root numbers (data not shown). At 0 d, 3 d, 6 d and 9 d, the concentrations of free proline and soluble sugars were investigated in the transgenic lines. Three transgenic lines had significantly higher proline concentrations and soluble sugars as compared to the WT (Figure 2).

### The Expression of Salt-responsive Genes in Transgenic Soybean

**Figure 3:**
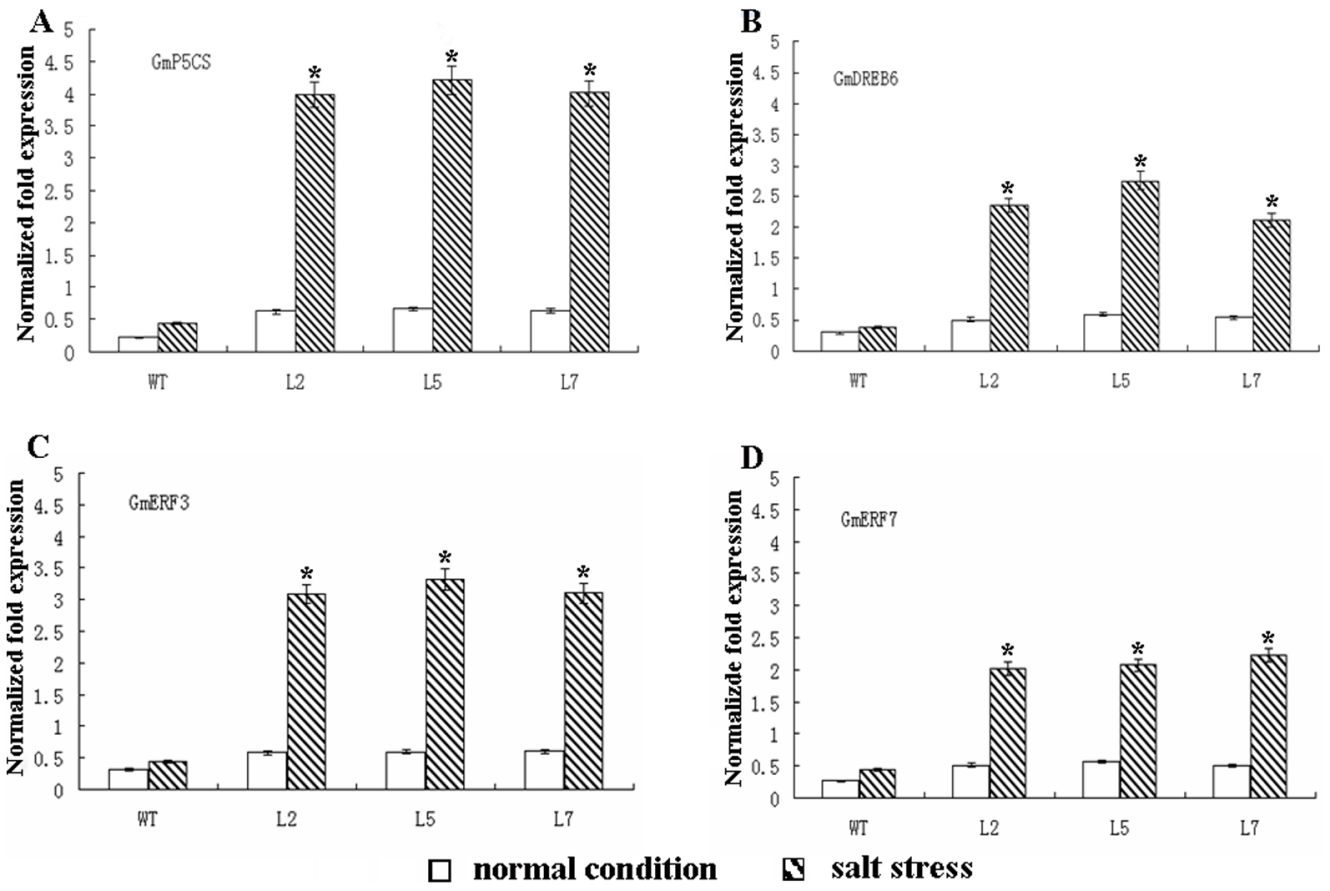
Expression patterns of salt stress-response genes. *Tubulin* was used as an internal control. Four genes, *GmP5CS*, *GmDREB6*, *GmERF3*, and *GmERF7* showed increased expression levels in the transgenic soybeans compared to the WT. Values are the mean of four biological replicates ± SE.

To elucidate the putative molecular mechanisms of *OsDREB2A* in the high-salt response, we investigated the expression of 10 abiotic stress-response genes, including *GmNHX1*, *GmDREB1*, *GmDREB3*, *GmDREB5*, *GmDREB6*, *GmERF3*, *GmERF7*, *Gmcor47-like*, *GmKin* and *GmP5CS* in both transgenic and WT plants under normal and 300 mM salt stress conditions, respectively. According to the qRT-PCR analyses, four genes, *GmDREB6*, *GmP5CS*, *GmERF3*, and *GmERF7* were shown to have significantly (*P*<0.05) increased expression in the transgenic soybean under normal and salt stress condition, especially, the increasing tendency of *GmP5CS* was the most obvious effect in the four genes (*P<*0.05) (Figure 3). The other six genes were not differentially expressed in the WT and transgenic soybeans (Data not shown).

### DNA-binding Activity Analysis of *OsDREB2A* using Gel Mobility Shift Assay

**Figure 4:**
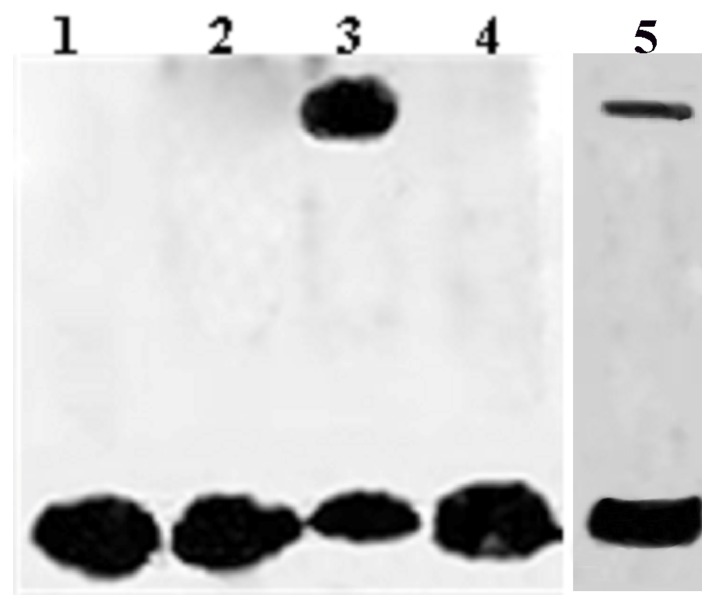
EMSA showing OsDREB2A specific binding to the DRE element. Lane 1, free labeled DRE probes; lane 2, GST proteins control; lane 3, GST-OsDREB2A fusion proteins plus labeled the WT DRE element; lane 4, GST-OsDREB2A fusion proteins plus labeled the mutant DRE element; and lane 5, GST-OsDREB2A fusion proteins plus unlabeled and labeled DRE element.

Gel mobility shift assay revealed that the DRE element could interact with *OsDREB2A*-GST fusion protein and was retarded on SDS-PAGE (Figure 4), the DNA-protein complex migrated more slowly than free DNA, which indicated that the *OsDREB2A* protein was able to specifically bind to the DRE element. In contrast, the *OsDREB2A* protein had no interaction with the mutated DRE element. These results suggested that OsDREB2A could specifically bind to DRE element containing -CCGAC-core sequence *in vitro*.

### Promoters of *OsDREB2A*-activated Genes Contain GCC and DRE *cis*-elements

It was previously shown that DREB is a trans-acting factor that can bind to the DRE/CRT(C-repeat) sequence which contains an A/GCCGAC motif to activate the gene expression in the stress-signaling pathway in plants. To determine whether promoters of *OsDREB2A*-activated genes contain putative *cis*-elements possibly binding to *OsDREB2A*, we searched for *cis*-elements in the 2-kb promoter region upstream of ATG in the PLACE database [25]. The promoter regions of *GmDREB6*, *GmERF3* and *GmERF7* all contain the *cis*-elements of GT-1 and DRE (Table 2). The *GmP5CS* promoter has GT-1, but no DRE. Although the promoter region of *GmDREB1* has one DRE *cis*-element (Table 2), it seems that *OsDREB2A* did not activate *GmDREB1* expression. Consistent with the *GmNHX1*, *GmDREB3*, *GmDREB5*, *Gmcor47-like* and *GmKin* genes expression, we did not find DRE in the promoter regions.

**Table 2:** *cis*-elements in promoters of OsDREB2A-activated genes.

Gene	ID	*cis*-elements
*GmNHX1*	Glyma10g30020	GT-1(−592, −817, −875, −1392)
*GmDREB1*	Glyma14g09320	DRE(−1085), GT-1(−213)
*GmDREB3*	Glyma17g05240	GT-1(−325, −633, −1012, −1120), GCC(−1610)
*GmDREB5*	Glyma12g33020	GT-1(−402, −705, −740, −1124)
*GmDREB6*	Glyma5g04920	DRE(−1113), GT-1(−133, −1398, −1488, −1560, −1993)
*GmERF3*	Glyma03g42440	DRE(−487), GT-1(−25, −1112, −1293, −1507, −1530, −1988)
*GmERF7*	Glyma07g04950	DRE(−1873), GT-1(−1782), G-box(−36)
*Gmcor47-like*	Glyma04g01130	GT-1(−779, −1700), GCC(−119)
*GmKin*	Glyma14g07880	GT-1(−80)
*GmP5CS*	Glyma18g03820	GT-1(−56, −1243, 1641), GCC(−1899)

## Discussion

DRE *cis-*acting element is mainly involved in regulation of genes by drought, salt and cold stress under ABA-independent pathways [26]. Previous studies have shown that DREB genes are resistant to different abiotic stresses, including salt, drought, oxidation, and freezing [12], [27], [28]. DREB2 are involved in drought and salt stresses but not in cold stress while DREB1/CBF-type transcription factors function in response to cold stress in another ABA-independent pathway [7], [11], [17], [29]. In the present study, our results showed that *OsDREB2A* overexpression improved high-salt tolerance in transgenic soybeans. And interestingly, *OsDREB2A* does not affect the developmental growth of soybean under normal growth conditions (Data not shown). Effect of salt stress on both seed germination and seedling growth showed that the transgenic lines increased tolerance to salt stress than the WT (Figure 1B, 1D and 1E). The results demonstrated that *OsDREB2A* acted as a positive regulator in response to salt stress at the germination and seedling stages in transgenic soybeans.

While many abiotic-stress-inducible genes are controlled by ABA, but some are not, which indicates that both ABA-dependent and ABA-independent regulatory pathways are involved in stress-responsive gene expression [30]. ABA has been suggested to be involved in the adaptation of plants to salt stress [31]. Cross-talk between salt and ABA signaling pathways has been demonstrated [6], [17], [32]. To investigate whether *OsDREB2A* is involved in ABA-dependant or independent salt-stress signaling, we determined the effect of ABA on seed germination and found it having a similar effect on the germination of both transgenic and WT seeds (Figure 1C), which suggested that *OsDREB2A* did not depend on ABA to improve plant salt tolerance in soybean. In this study, overexpression of *OsDREB2A* in soybean increased tolerance to salt and osmotic stress in an ABA-independent manner.

Previous studies have found that plants may enhance stress tolerance by accumulating osmolytes, such as soluble sugars and free proline to adjust the osmotic potential and protect cell structures [33], [34]. Our results showed that the contents of free proline and soluble sugars in the transgenic lines were higher than that in WT plants under high-salt stress (Figure 2), suggesting that *OsDREB2A* can regulate free proline and soluble sugars biosynthesis. Consistent with our results, previous studies have shown that overexpression of DREB family genes could elevate proline content [33], [35]–[37]. Indeed, it is well known that proline accumulation can increase the osmotic pressure, thus, improve the salt tolerance of plants [38].

Overexpression of *P5CS* also increased stress tolerance of transgenic potato, rice and wheat as a result of the increased proline content [39]–[42]. We analyzed the expression of *GmP5CS*, the key enzyme in proline synthesis [43], [44], and some other genes known to be related to salt resistance. Similarly, qRT-PCR analysis revealed that the expression of *OsDREB2A* increased *GmP5CS* transcript level and thus led to higher level of free proline accumulation (Figures 2 and 3). Promoter analysis of the *GmP5CS* revealed that no DRE region exists. The interaction of *OsDREB2A* with *GmP5CS* promoter must not contribute to the increased expression of *GmP5CS*. With the discovery of miRNAs, a new mechanism to regulate protein expression has been revealed. Considering the inconsistency between *P5CS* mRNA and protein expression and the importance of miRNAs in cancer, the regulation of miRNAs on *P5CS* represent a very promising hypothesis [45]. In this study, we propose that *OsDREB2A* might be involved in many pathways, in addition to combination with DRE region, including regulation of miRNAs and other pathways. A lot of efforts are still required to uncover in detail of each products of gene induced by *P5CS* and their interacting partners to understand the complexity of the high salinity stress signal transduction pathways.

In order to dissect the enhanced salt tolerance at the molecular level, expression of 10 stress-responsive genes were monitored between the transgenic soybeans and WT. Our results showed that four out of ten, such as *GmDREB6*, *GmP5CS*, *GmERF3*, and *GmERF7* were higher expression in transgenic soybeans compared to WT (Figure 3).

We inferred that *OsDREB2A* might act as an activator to increase the expression of the above stress responsive genes to enhance tolerance of the transgenic soybeans under salt stress. The EMSA indicated that OsDREB2A can bind to DRE. However, it is unknown if OsDREB2A has other binding specificities (Figure 4). Although the promoter of *GmDREB1* has one DRE *cis*-elements (Table 2), it seems that *OsDREB2A* did not activate *GmDREB1* expression (Data not shown). It is not clear why it occurs.

In conclusion, overexpression of *OsDREB2A* led to the up-regulated expression of several key stress responsive genes, increased accumulation of soluble sugars and free proline, and enhanced tolerance to salt stress in transgenic soybean. Our data suggested that *OsDREB2A* probably functions as a crucial positive transcription factor in the complex regulatory systems for salt stress response. These results may provide valuable insight into the role of *OsDREB2A* in abiotic stress tolerance of different species.
